# What a Dentist Has Done

**Published:** 1890-12

**Authors:** 


### What a Dentist Has Done.

As is well known dentists will sometimes step outside of the field of their own specialty and make themselves useful to the world in other directions as well as in dental practice. An illustration of this is presented by Dr. Eli Collins, D.D.S., of Little Rock, Arkansas, formerly of Cincinnati, O.

The doctor was always of an inventive turn of mind. A number of devices and inventions have been the product of his genius. The recent, and perhaps the most important of all his inventions is a "*Car heating apparatus*" for which he has received a patent. This is a device and apparatus which can not be explained here, but the intent is to heat and ventilate a train of cars without the possibility of an accident by fire. Both of these objects seem to be most perfectly accomplished, namely: thorough ventilation and sufficient heat. The many fatal casualties by fire in wrecked trains fully emphasizes the value of such a device. If such an invention has been attained as will absolutely accomplish these objects, as seems to be the case with this one, no train that requires heating should be allowed to run with the dangerous heating apparatus heretofore employed. If a safe heating apparatus has been found every train in the land should be compelled by law to use it. The safety of the traveling public and humanity generally demand this. Every State in the Union should at once enact most stringent laws regulating this subject. The man who succeeds in bringing forth a device of this kind is a benefactor to his race.

